# Supplementary material for: Individual retrotransposon integrants are differentially controlled by KZFP/KAP1-dependent histone methylation, DNA methylation and TET-mediated hydroxymethylation in naïve embryonic stem cells
Source: Epigenetics Chromatin. 2018 Feb 26;11:7. doi: 10.1186/s13072-018-0177-1 (PMC6389204; doi:10.1186/s13072-018-0177-1)
Supplement: Supplementary file 11 — Additional file 11. Pattern analysis. [file 13072_2018_177_MOESM11_ESM.zip › Patterns analysis/DataTables/extensions/Responsive/examples/child-rows/whole-row-control.html]

Responsive example - Whole row child row control


# Responsive example Whole row child row control

When using the `column` details type in Responsive the `responsive.details.targetR`
option provides the ability to control what element is used to show / hide the child rows when the
table is collapsed.

This example uses the `tr` selector to have the whole row act as the control.

|  | First name | Last name | Position | Office | Age | Start date | Salary | Extn. |
| --- | --- | --- | --- | --- | --- | --- | --- | --- |
|  | First name | Last name | Position | Office | Age | Start date | Salary | Extn. |
| --- | --- | --- | --- | --- | --- | --- | --- | --- |
|  | Tiger | Nixon | System Architect | Edinburgh | 61 | 2011/04/25 | $320,800 | 5421 |
|  | Garrett | Winters | Accountant | Tokyo | 63 | 2011/07/25 | $170,750 | 8422 |
|  | Ashton | Cox | Junior Technical Author | San Francisco | 66 | 2009/01/12 | $86,000 | 1562 |
|  | Cedric | Kelly | Senior Javascript Developer | Edinburgh | 22 | 2012/03/29 | $433,060 | 6224 |
|  | Airi | Satou | Accountant | Tokyo | 33 | 2008/11/28 | $162,700 | 5407 |
|  | Brielle | Williamson | Integration Specialist | New York | 61 | 2012/12/02 | $372,000 | 4804 |
|  | Herrod | Chandler | Sales Assistant | San Francisco | 59 | 2012/08/06 | $137,500 | 9608 |
|  | Rhona | Davidson | Integration Specialist | Tokyo | 55 | 2010/10/14 | $327,900 | 6200 |
|  | Colleen | Hurst | Javascript Developer | San Francisco | 39 | 2009/09/15 | $205,500 | 2360 |
|  | Sonya | Frost | Software Engineer | Edinburgh | 23 | 2008/12/13 | $103,600 | 1667 |
|  | Jena | Gaines | Office Manager | London | 30 | 2008/12/19 | $90,560 | 3814 |
|  | Quinn | Flynn | Support Lead | Edinburgh | 22 | 2013/03/03 | $342,000 | 9497 |
|  | Charde | Marshall | Regional Director | San Francisco | 36 | 2008/10/16 | $470,600 | 6741 |
|  | Haley | Kennedy | Senior Marketing Designer | London | 43 | 2012/12/18 | $313,500 | 3597 |
|  | Tatyana | Fitzpatrick | Regional Director | London | 19 | 2010/03/17 | $385,750 | 1965 |
|  | Michael | Silva | Marketing Designer | London | 66 | 2012/11/27 | $198,500 | 1581 |
|  | Paul | Byrd | Chief Financial Officer (CFO) | New York | 64 | 2010/06/09 | $725,000 | 3059 |
|  | Gloria | Little | Systems Administrator | New York | 59 | 2009/04/10 | $237,500 | 1721 |
|  | Bradley | Greer | Software Engineer | London | 41 | 2012/10/13 | $132,000 | 2558 |
|  | Dai | Rios | Personnel Lead | Edinburgh | 35 | 2012/09/26 | $217,500 | 2290 |
|  | Jenette | Caldwell | Development Lead | New York | 30 | 2011/09/03 | $345,000 | 1937 |
|  | Yuri | Berry | Chief Marketing Officer (CMO) | New York | 40 | 2009/06/25 | $675,000 | 6154 |
|  | Caesar | Vance | Pre-Sales Support | New York | 21 | 2011/12/12 | $106,450 | 8330 |
|  | Doris | Wilder | Sales Assistant | Sidney | 23 | 2010/09/20 | $85,600 | 3023 |
|  | Angelica | Ramos | Chief Executive Officer (CEO) | London | 47 | 2009/10/09 | $1,200,000 | 5797 |
|  | Gavin | Joyce | Developer | Edinburgh | 42 | 2010/12/22 | $92,575 | 8822 |
|  | Jennifer | Chang | Regional Director | Singapore | 28 | 2010/11/14 | $357,650 | 9239 |
|  | Brenden | Wagner | Software Engineer | San Francisco | 28 | 2011/06/07 | $206,850 | 1314 |
|  | Fiona | Green | Chief Operating Officer (COO) | San Francisco | 48 | 2010/03/11 | $850,000 | 2947 |
|  | Shou | Itou | Regional Marketing | Tokyo | 20 | 2011/08/14 | $163,000 | 8899 |
|  | Michelle | House | Integration Specialist | Sidney | 37 | 2011/06/02 | $95,400 | 2769 |
|  | Suki | Burks | Developer | London | 53 | 2009/10/22 | $114,500 | 6832 |
|  | Prescott | Bartlett | Technical Author | London | 27 | 2011/05/07 | $145,000 | 3606 |
|  | Gavin | Cortez | Team Leader | San Francisco | 22 | 2008/10/26 | $235,500 | 2860 |
|  | Martena | Mccray | Post-Sales support | Edinburgh | 46 | 2011/03/09 | $324,050 | 8240 |
|  | Unity | Butler | Marketing Designer | San Francisco | 47 | 2009/12/09 | $85,675 | 5384 |
|  | Howard | Hatfield | Office Manager | San Francisco | 51 | 2008/12/16 | $164,500 | 7031 |
|  | Hope | Fuentes | Secretary | San Francisco | 41 | 2010/02/12 | $109,850 | 6318 |
|  | Vivian | Harrell | Financial Controller | San Francisco | 62 | 2009/02/14 | $452,500 | 9422 |
|  | Timothy | Mooney | Office Manager | London | 37 | 2008/12/11 | $136,200 | 7580 |
|  | Jackson | Bradshaw | Director | New York | 65 | 2008/09/26 | $645,750 | 1042 |
|  | Olivia | Liang | Support Engineer | Singapore | 64 | 2011/02/03 | $234,500 | 2120 |
|  | Bruno | Nash | Software Engineer | London | 38 | 2011/05/03 | $163,500 | 6222 |
|  | Sakura | Yamamoto | Support Engineer | Tokyo | 37 | 2009/08/19 | $139,575 | 9383 |
|  | Thor | Walton | Developer | New York | 61 | 2013/08/11 | $98,540 | 8327 |
|  | Finn | Camacho | Support Engineer | San Francisco | 47 | 2009/07/07 | $87,500 | 2927 |
|  | Serge | Baldwin | Data Coordinator | Singapore | 64 | 2012/04/09 | $138,575 | 8352 |
|  | Zenaida | Frank | Software Engineer | New York | 63 | 2010/01/04 | $125,250 | 7439 |
|  | Zorita | Serrano | Software Engineer | San Francisco | 56 | 2012/06/01 | $115,000 | 4389 |
|  | Jennifer | Acosta | Junior Javascript Developer | Edinburgh | 43 | 2013/02/01 | $75,650 | 3431 |
|  | Cara | Stevens | Sales Assistant | New York | 46 | 2011/12/06 | $145,600 | 3990 |
|  | Hermione | Butler | Regional Director | London | 47 | 2011/03/21 | $356,250 | 1016 |
|  | Lael | Greer | Systems Administrator | London | 21 | 2009/02/27 | $103,500 | 6733 |
|  | Jonas | Alexander | Developer | San Francisco | 30 | 2010/07/14 | $86,500 | 8196 |
|  | Shad | Decker | Regional Director | Edinburgh | 51 | 2008/11/13 | $183,000 | 6373 |
|  | Michael | Bruce | Javascript Developer | Singapore | 29 | 2011/06/27 | $183,000 | 5384 |
|  | Donna | Snider | Customer Support | New York | 27 | 2011/01/25 | $112,000 | 4226 |

- Javascript
- HTML
- CSS
- Ajax
- Server-side script

The Javascript shown below is used to initialise the table shown in this
example:

`$(document).ready(function() {
$('#example').DataTable( {
responsive: {
details: {
type: 'column',
target: 'tr'
}
},
columnDefs: [ {
className: 'control',
orderable: false,
targets: 0
} ],
order: [ 1, 'asc' ]
} );
} );`

In addition to the above code, the following Javascript library files are loaded for use in this
example:

- ../../../../media/js/jquery.js
- ../../../../media/js/jquery.dataTables.js
- ../../js/dataTables.responsive.js

The HTML shown below is the raw HTML table element, before it has been enhanced by
DataTables:

This example uses a little bit of additional CSS beyond what is loaded from the library
files (below), in order to correctly display the table. The additional CSS used is shown
below:

The following CSS library files are loaded for use in this example to provide the styling of the
table:

- ../../../../media/css/jquery.dataTables.css
- ../../css/dataTables.responsive.css

This table loads data by Ajax. The latest data that has been loaded is shown below. This data
will update automatically as any additional data is loaded.

The script used to perform the server-side processing for this table is shown below. Please note
that this is just an example script using PHP. Server-side processing scripts can be written in any
language, using the protocol described in the
DataTables documentation.

## Other examples

### Basic initialisation

- Class name
- Configuration option
- `new` constructor
- Ajax data
- Default initialisation

### Styling

- Bootstrap styling
- Foundation styling

### Display control

- Automatic column hiding
- Class control
- Assigned class control

### Child rows

- Disable child rows
- Column controlled child rows
- Column control - right
- Whole row child row control
- Custom child row renderer

Please refer to the DataTables documentation for full
information about its API properties and methods.  
Additionally, there are a wide range of extras and
plug-ins which extend the capabilities of
DataTables.

DataTables designed and created by SpryMedia Ltd © 2007-2014  
DataTables is licensed under the MIT license.
